# Temporal analysis of prevalence and antibiotic-resistance patterns in *Stenotrophomonas maltophilia* clinical isolates in a 19-year retrospective study

**DOI:** 10.1038/s41598-024-65509-z

**Published:** 2024-06-24

**Authors:** Meshal K. AlFonaisan, Murad A. Mubaraki, Sahar I. Althawadi, Dalia A. Obeid, Ahmed A. Al-Qahtani, Reem S. Almaghrabi, Fatimah S. Alhamlan

**Affiliations:** 1 Department of Infection and Immunity, King Faisal Specialist Hospital and Research Centre, P.O.BOX 3354, 11211 Riyadh, Saudi Arabia; 2 Faculty Member, Majmaah University, Al Majmaah, Saudi Arabia; 3 Clinical Laboratory Sciences Department, College of Applied Medical Sciences, King Saud University, Riyadh, Saudi Arabia; 4 Department of Pathology and Laboratory Medicine, King Faisal Specialist Hospital and Research Centre, Riyadh, Saudi Arabia; 5 Organ Transplant Centre of Excellence, King Faisal Specialist Hospital and Research Centre, Riyadh, Saudi Arabia; 6 College of Medicine, Alfaisal University, Riyadh, Saudi Arabia

**Keywords:** Antimicrobials, Bacteriology, Infectious diseases

## Abstract

*Stenotrophomonas maltophilia* is a nonfermenting gram-negative bacterium associated with multiple nosocomial outbreaks. Antibiotic resistance increases healthcare costs, disease severity, and mortality. Multidrug-resistant infections (such as *S. maltophilia* infection) are difficult to treat with conventional antimicrobials. This study aimed to investigate the isolation rates, and resistance trends of *S. maltophilia* infections over the past 19 years, and provide future projections until 2030. In total, 4466 patients with *S. maltophilia* infection were identified. The adult and main surgical intensive care unit (ICU) had the highest numbers of patients (32.2%), followed by the cardiology department (29.8%), and the paediatric ICU (10%). The prevalence of *S. maltophilia* isolation increased from 7% [95% confidence interval (CI) 6.3–7.7%] in 2004–2007 to 15% [95% CI 10.7–19.9%] in 2020–2022. Most *S. maltophilia* isolates were resistant to ceftazidime (72.5%), levofloxacin (56%), and trimethoprim-sulfamethoxazole (14.05%), according to our study. A consistent and significant difference was found between *S. maltophilia*-positive ICU patients and non-ICU patients (P = 0.0017) during the three-year pandemic of COVID-19 (2019–2021). The prevalence of *S. maltophilia* isolates is expected to reach 15.08% [95% CI 12.58–17.59%] by 2030. Swift global action is needed to address this growing issue; healthcare authorities must set priorities and monitor infection escalations and treatment shortages.

## Introduction

*Stenotrophomonas maltophilia* is a nonfermenting gram-negative bacillus^1^ found in several environmental reservoirs, including plants, soil, and animals^2^. In addition, it is widely distributed in the environment as a commensal organism, and its impact on severe diseases has been observed on a global scale^1^. It is a pathogenic microorganism responsible for nosocomial infections in individuals with compromised immune systems, specifically healthcare-associated pneumonia in the intensive care unit (ICU) settings^3,4^. Hospital water pipes, sinkholes, and catheters are potential sources of disease transmission^1^. From a clinical perspective, *S. maltophilia* causes a variety of diseases, including respiratory tract infection (pneumonia), bone and joint infection, bloodstream infection (BSI), meningitis, and urinary tract infection (UTI)^5,6^. Most *β*-lactams, fluoroquinolones, aminoglycosides, and trimethoprims are ineffective against *S. maltophilia* because of the bacterium’s intrinsic resistance to these classes of antibiotics. Managing infections caused by *S. maltophilia* is complicated by the fact that the organism is resistant to many antibiotics^7^, a trait known as multidrug resistance (MDR). Furthermore, various clinical laboratories have reported the emergence of pandrug-resistant (PDR) strains of *S. maltophilia*. These strains are resistant to all available antimicrobial medications. This might be attributed to the improper use of antibiotics, especially broad-spectrum antibiotics^8,9^. *S. maltophilia* infections are often treated with trimethoprim-sulfamethoxazole (SXT); levofloxacin (LVX) may be used as a secondary option. Ceftazidime (CAZ) and ticarcillin-clavulanate (TIM) are potential therapeutic options. Nonetheless, the previous use of antibiotics may hasten the development of resistant bacteria^10,11^.

However, an increase in the incidence of MDR bacteria, particularly *S. maltophilia*, has been documented among hospitalised coronavirus disease 2019 (COVID-19) patients owing to the overuse of antimicrobial agents during the severe acute respiratory syndrome coronavirus 2 (SARS-CoV-2) pandemic, which emerged in China in 2019^12,13^. The resistance characteristics of *S. maltophilia* in Saudi Arabia and the neighbouring region have been the subject of limited research amidst the COVID-19 pandemic. In light of this, the current study aimed to examine the historical pattern of *S. maltophilia* detection and resistance trends in the last 19 years (2004–2022) and investigate the forecast of infections until 2030.

## Results

### Descriptive analysis of the data collected

A total of 4466 patient records of *S. maltophilia* infections were obtained. The adult and main surgical ICU (AD/MSICU) had the highest proportion of reported cases (32.2%), followed by a combination of other departments including but not limited to family medicine clinics, eye clinic, orthopaedics Clinics, immunology diseases clinic, nephrology clinic, colorectal clinic, and otolaryngology clinic (29.8%), and the cardiology ward (15.9%). An estimated 10.8% of the patients were reported in the paediatric ICU (Table 1). The number of samples tested was lowest between 2004 and 2007. In 2021, the largest number of samples collected (9.5%), followed by 2019 (7.6%). From 2004 to 2007, the prevalence of *S. maltophilia* infection was 7% [95% confidence interval [CI] 6.3–7.7%], withal, by 2020–2022, the prevalence reached 15% [95% CI 10.7–19.9%]. The highest prevalence rate occurred between 2016 and 2019 with 20% [95% CI 15–25.2%].

**Table 1:** Distribution of *S. maltophilia* isolates among different hospital wards.

Department/medical ward	Count, N	(%)
Adult and surgical ICUs	1436	32.2
Others^a^	1333	29.8
Cardiology ward	712	15.9
Paediatric ICU	481	10.8
Emergency department	107	2.4
Adult haematology/oncology wards	87	1.9
Paediatric haematology/oncology wards	77	1.7
King Abdullah Centre for Oncology & Liver Diseases	67	1.5
Adult outpatient department	61	1.4
Adult surgical wards	48	1.1
Adult medical wards	47	1.1
Paediatric medical wards	7	0.2
Paediatric outpatient department	3	0.1
Paediatric surgical wards	0	0.0
Neonatal ICU	0	0.0
Gynaecology and obstetrics ward	0	0.0

^a^Other hospital departments.

### Antibiotic susceptibility of *S. maltophilia* isolates

**Figure 1:**
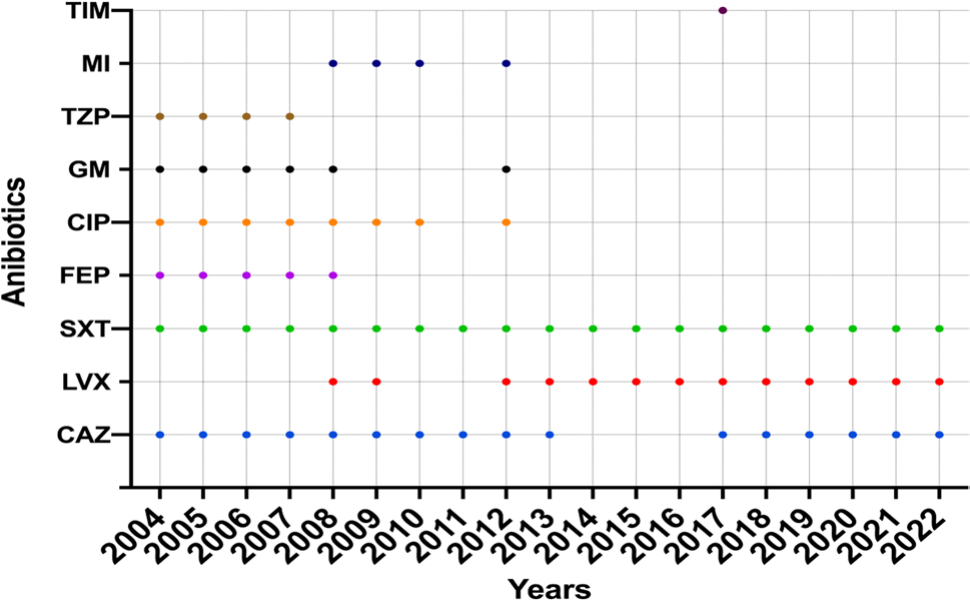
Antibiotic accessibility in 2004–2022. *CAZ* ceftazidime, *LVX* levofloxacin, *SXT* trimethoprim-sulfamethoxazole, *FEP* cefepime, *CIP* ciprofloxacin, *GM* gentamicin, *TZP* piperacillin-tazobactam, *MI* minocycline, *TIM* ticarcillin-clavulanate.

**Figure 2:**
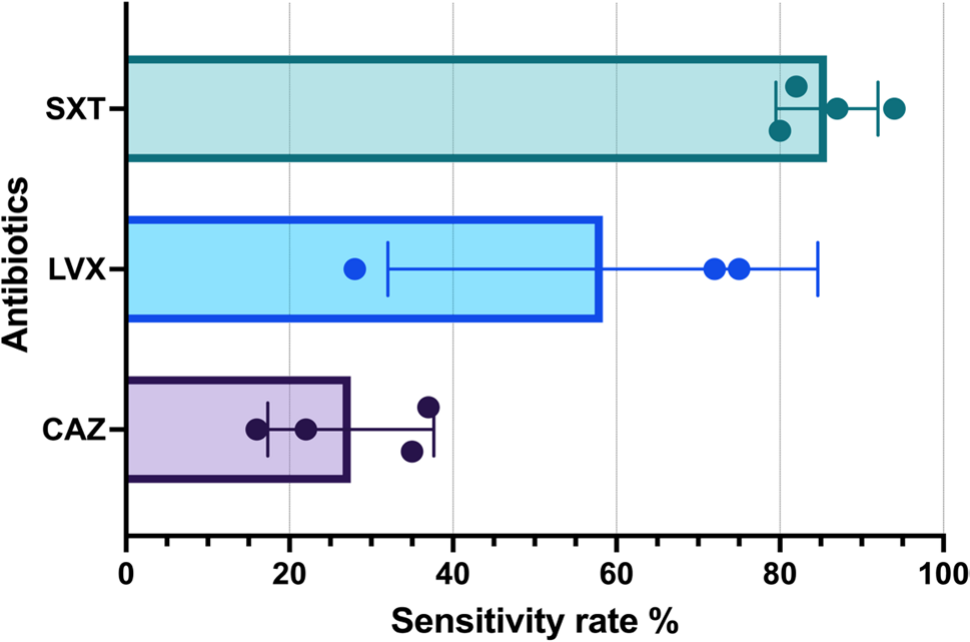
Bar graph of the antimicrobial sensitivity test results in 2004–2022. Dots represents four periods of antibiotic susceptibility: 2004–2008, 2009–2013, 2014–2018, and 2019–2022. Note that LVX was not tested during the first period. *CAZ* ceftazidime, *LVX* levofloxacin, *SXT* trimethoprim-sulfamethoxazole.

**Figure 3:**
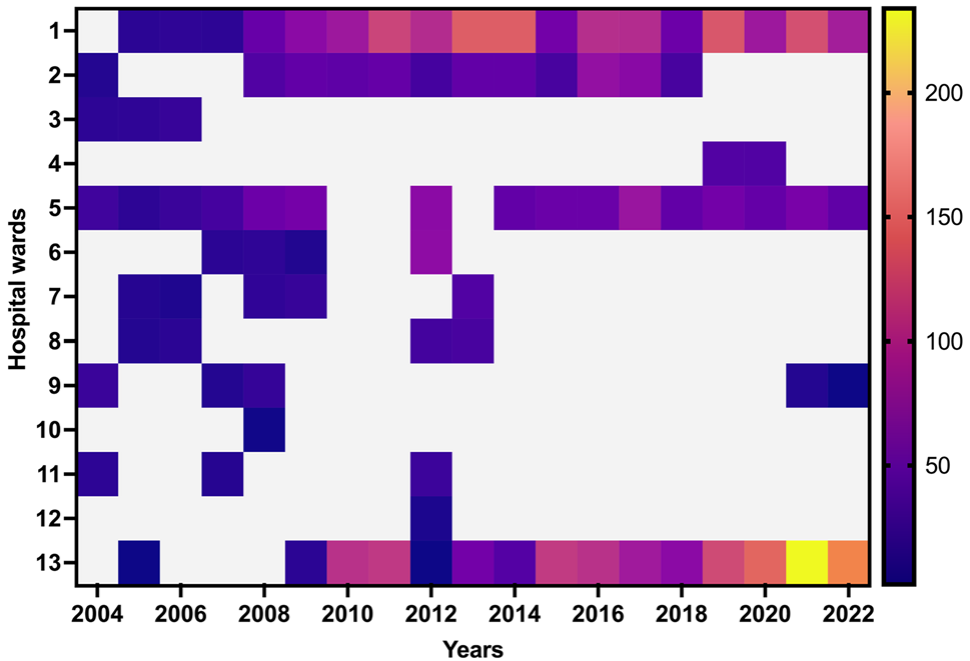
Heatmap illustrating the consumption of antibiotics across 13 different hospital wards throughout the time span of 2004–2022. 1: Adult ICUs; 2: Paediatric ICUs; 3: Surgical wards; 4: King Abdullah Centre for Oncology & Liver Diseases; 5: Cardiology wards; 6: Emergency department; 7: Adult haematology/oncology wards; 8: Paediatric haematology/oncology wards; 9: Adult out-patient department; 10: Paediatric out-patient department; 11: Adult medical wards; 12: Paediatric medical wards; 13: Others (other additional medical departments.).

The criteria used for the interpretation of the susceptibility test results adhered to the guidelines established by the CLSI. Table 2 presents the distribution of susceptibility testing findings according to the antibiotics used, categorised by year and the related number of cases. However, except for SXT, most antibiotics were not consistently accessible over all 19 years. Ceftazidime was accessible in all years, except in 2014, 2015, and 2016. LVX was accessible for the entire duration, except in 2004–2007, 2010, and 2011. TIM was administered only in 2017 (Fig. 1). The average susceptibility rates of *S. maltophilia* within 19 years were the highest for SXT (85.7%), followed by LVX (44%), and ceftazidime (27.6%) (Fig. 2). Notably, in vitro analysis of SXT revealed a progressive decline in the average susceptibility from 94 to 80% in 2004–2022. The efficacy of MI and other antibiotics was only tested against MDR isolates (Fig. 3)^14^.

**Table 2:** The overall susceptibility rate of selected antibiotics that have been used from 2004 to 2022. Each value in the cell represents the mean susceptibility rate and the range (max–min). *Please note that not all antibiotics were accessible during the 19-year period. *NT* not tested. **Percentage of the total collected samples (4466).

Overall isolate counts and AST results*						
#	Year	Count (total)	%**	Ceftazidime	Levofloxacin	Trimethoprim-sulfamethoxazole
1	2004–2008	479	10.73	19.50 (41.00–4.00)	NT	93.80 (100.00–83.00)
2	2009–2013	1306	29.24	35.2 (48.00–23.00)	71.00 (79.00–63.00)	87.00 (93.00–77.00)
3	2014–2018	1324	29.65	37.33 (54.00–33.00)	75.20 (81.00–70.00)	81.80 (90.00–73.00)
4	2019–2022	1357	30.39	49.33 (62.00–27.00)	72.25 (79.00–68.00)	80.00 (83.00–77.00)

### Effects of COVID-19 pandemic on *S. maltophilia* infections

**Figure 4:**
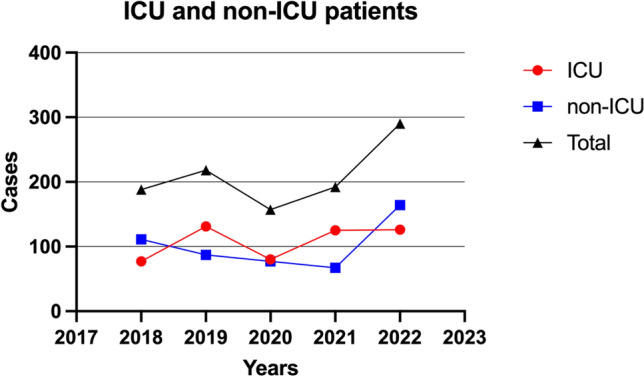
The incidence of *S. maltophilia* infections among patients in both ICU and non-ICU settings, with a specific focus on the observed increase in *S. maltophilia* detection rates during the COVID-19 pandemic (2019, 2020, and 2021) in comparison to the rates seen prior and after the pandemic.

The rates of *S. maltophilia* infection were studied in ICU and non-ICU patients. The overall number of admissions before and during the COVID-19 pandemic was also determined. A significant difference was found between the proportion of ICU patients with positive *S. maltophilia* cultures and that of non-ICU patients (P = 0.0017) within 3-years, including 2019, 2020, and 2021 (Fig. 4). Furthermore, a statistically significant difference was seen between the numbers of ICU and non-ICU *S. maltophilia*-positive isolates in the years 2019 and 2021 (P = 0.0372 and P = 0.0033, respectively).

It is noteworthy that the number of non-ICU patients with *S. maltophilia* isolates before and after the pandemic (in 2018 and 2022, respectively) exceeded the number of ICU patients with *S. maltophilia* isolates in the same period.

### Current trend and future forecast

**Figure 5:**
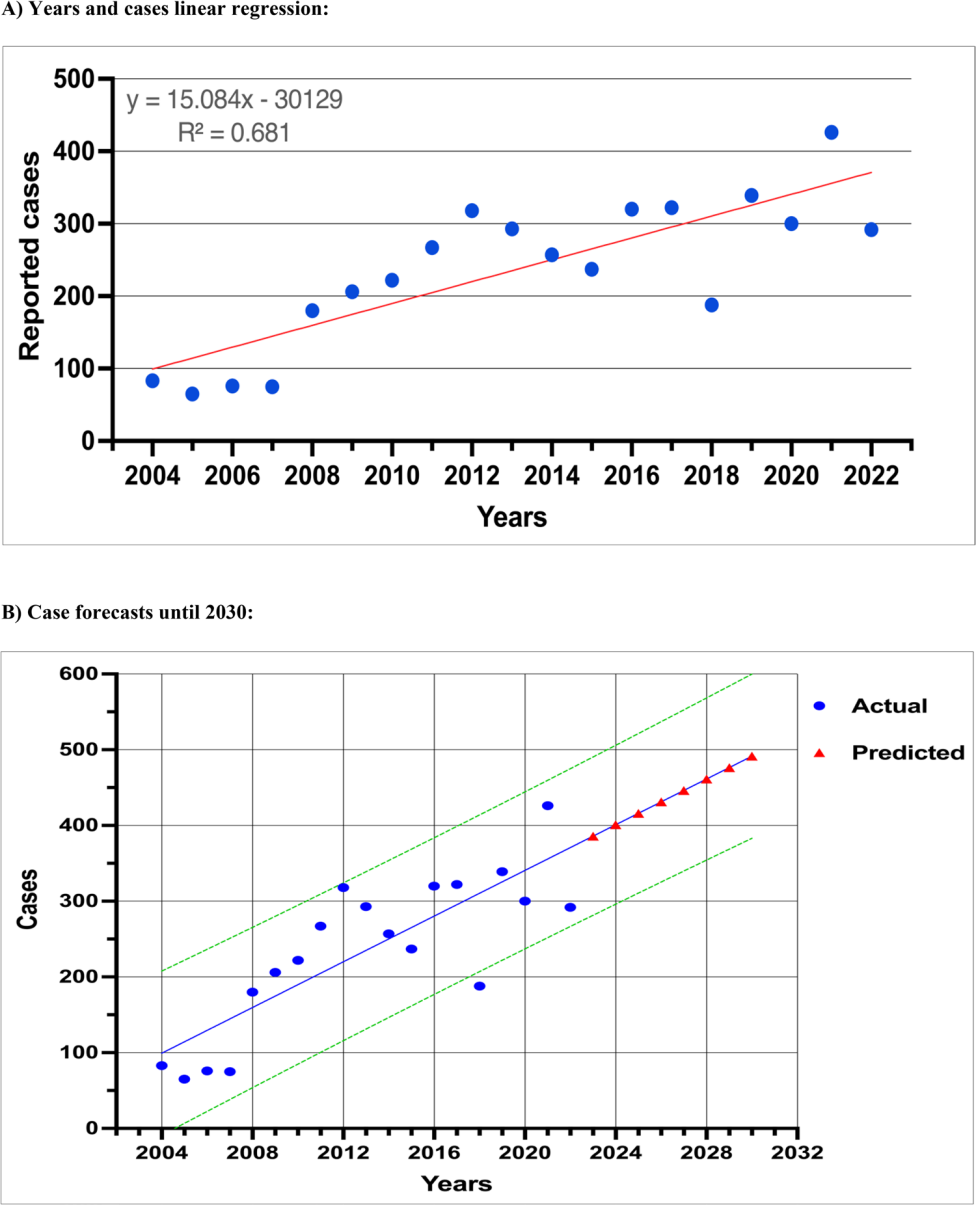
(**a**) Significant linear regression of *S. maltophilia* cases identified from 2004 to 2022. (**b**) The forecast model, developed using the Prophet package in the R program, gives a projection of forthcoming instances until 2030, revealing an upward trend in the number of cases.

One of the objectives of this study is to use linear regression analysis in order to investigate the chronological patterns of *S. maltophilia* cases identified throughout the period from 2004 to 2022. The results of the linear regression analysis indicated a statistically significant positive correlation, as shown by the strong coefficient (R^2^ = 0.65, P < 0.001). The forecast model, developed using the Prophet package inside the R programming language shows a projection of forthcoming instances, demonstrating a discernible rising trajectory in the number of cases (Fig. 5).

## Discussion

According to the available data, this study presents the most extensive dataset on the antimicrobial resistance rates (AMRs) of *S. maltophilia* ever documented in Saudi Arabia and its neighbouring regions. Recently, the detection rate of *S. maltophilia* within healthcare facilities has continuously increased^15^. Hospital-acquired infections (HAI) caused by *S. maltophilia* have also been on an increase, particularly in immunocompromised individuals^2,5^. Consequently, there is a growing need to comprehensively investigate into the risk factors associated with this pathogen, prompting concerns within the medical community. Moreover, relatively few studies have been conducted on *S. maltophilia* clinical isolates in Saudi Arabia before to and during the COVID-19 pandemic, and this is one of them.

Therefore, this retrospective study conducted a comprehensive analysis over a period of 19 years, including 4466 patients with *S. maltophilia*. The current study collected clinical data from several hospital wards, with the majority of the study patients originating in the ICUs, followed by the cardiology wards. These results are not unforeseen; given that previous studies conducted in KFMC in Riyadh (2022) and KKUH (2012) have similarly reported the majority of *S. maltophilia* isolates were from patients admitted in the ICUs^16,17^. By contrast, our results contradicted the findings of the KFMC (2022)^17^ study and showed a significant correlation between the prevalence of *S. maltophilia* isolates between ICU and non-ICU patients before and during the COVID-19 pandemic. The increased rates of *S. maltophilia* isolates during the COVID-19 pandemic could be attributed to factors such as prolonged hospital stays that have been associated with a higher risk of *S. maltophilia* infections, broad-spectrum antibiotic use which has been linked to the selection of this pathogen, mechanical ventilation, and the use of invasive medical devices. Therefore, stringent infection control measures and judicious use of antibiotics are crucial to mitigate this risk^15,18,19^. To the best of our knowledge, no studies have been conducted comparing the prevalence of *S. maltophilia* infections among ICU patients before and after the COVID-19 pandemic. Furthermore, nearly all of global studies have focused on examining the prevalence of bacterial co-infections in ICUs among individuals with COVID-19. Notably, there has been a lack of research comparing the prevalence of these infections before and after the pandemic. The incidence of HAI caused by *S. maltophilia* may have been reduced owing to the implementation of infection control measures in healthcare facilities during the COVID-19 pandemic^20^. The analysis throughout the period of 2004–2022 showed a notable upward trend in the prevalence of *S. maltophilia* isolates. Specifically, the prevalence rate increased from 7% [95% confidence interval [CI] 6.3–7.7%] in 2004–2007 to 15% [95% CI 10.7–19.9%] in 2020–2022. The period from 2016 to 2019 had the highest prevalence rate, estimated at 20% [95% CI 15–25.2%]. The increasing prevalence of the isolation of this bacterium is consistent with global reports identifying it as an emerging opportunistic pathogen^15,20,21^. This trend may be attributed to the advancements in detection methods, the susceptibility of immunocompromised patients to *S. maltophilia* infections, and its ability to exhibit resistance to a broad spectrum of antimicrobial agents. From a global standpoint, *S. maltophilia* is a major contributor to infections in ICU patients, as evidenced by research conducted in Greece and Spain in 2023^22^, which included 103 non-COVID-19 patients. Likewise, a systematic review conducted in 2022^23^ further confirmed the prevalence of this pathogen among ICU patients.

Equally important, according to the World Health Organisation (WHO), *S. maltophilia* is one of the most common MDR pathogens found in healthcare facilities^24^. The use of SXT and LVX has been widely regarded as the primary approach for antimicrobial therapy in cases of *S. maltophilia* infections^25^. Due to the remarkable grade of intrinsic or/and acquired antibacterial resistance in *S. maltophilia*, therapeutic options are limited^26^. Our study revealed a high prevalence of resistance among *S. maltophilia* isolates towards ceftazidime (72.5%), LVX (56%), and SXT (14.05%). In a study conducted at the KFMC in 2022, 62.1% of *S. maltophilia* isolates exhibited resistance to ceftazidime. In addition, 14.8% of the isolates were resistant to LVX, whereas 4.1% were resistant to SXT. In another study conducted at KKUH in 2012, 57.21% of *S. maltophilia* isolates were resistant to ceftazidime, whereas 9.45% were resistant to SXT. The findings presented in this study suggest a notable rise in the ceftazidime resistance rate, perhaps attributable to the widespread and prolonged use of this antibiotic in recent years. Propitiously, SXT continues to demonstrate efficacy as an empirical treatment for infections caused by *S. maltophilia*. However, the increasing prevalence of resistance to SXT, which has traditionally been the preferred medication for treating *S. maltophilia* infections, is a matter of worry. Similarly, in a study conducted between 2006 and 2016, the resistance of 130 *S. maltophilia* isolates to SXT was examined in several centres in the United States. The results revealed resistance rates, ranging from 4 to 21%. The rate of resistance to ceftazidime throughout the specified period ranged from 57 to 84%^25^. A meta-analysis revealed that the areas outside the Eastern Mediterranean Region (EMR) and the Americas Region (AMR) reported the greatest global rates of resistance to SXT, reaching 20%. Comparatively, the resistance rate of EMR was 4.5%, whereas that of AMR was 13.1%. Additionally, ceftazidime has a substantial worldwide resistance rate of 65.1%, which is consistent with the findings of our study^14^. Moreover, when taken alongside other antibiotics, ceftazidime is an effective therapy for infections caused by *S. maltophilia*^27^. Likewise, a separate systematic review showed that the prevalence rates of *S. maltophilia* resistance to SXT were 43.82% in Asia, 30.33% in Europe, 23.59% in the Americas, and 2.24% in Africa. In comparison, the rates of resistance to LVX were 44.11%, 26.47%, 27.94%, and 1.47% in the same regions, respectively^21^. The same study reported that the global prevalence rates of LVX and SXT resistance were 14.4% and 9.2%, respectively. Concerningly, the rate of SXT resistance observed in our study was 14.05%, which surpasses both the global rate and China’s rate of 14.03%, the latter being the highest reported worldwide^20^. On the contrary, Africa had the lowest *S. maltophilia* antibiotic resistance rates, perhaps owing to the fewer isolates were identified and/or tested^15,21^. One potential factor contributing to the increased resistance rates of *S. maltophilia* to LVX is the similarity in the efficacy of LVX and SXT, along with a comparatively lower incidence of adverse effects associated with LVX^15^. This favourable risk-benefit profile positions LVX as the preferred treatment for many patients, hence increasing its likelihood of being overused or misused. Notably, most regional clinical laboratories use automated instruments to determine the minimum inhibitory concentrations (MICs) of SXT, which, according to certain studies, were less accurate compared with alternative approaches, such as disc diffusion. Thus, further investigation is required to establish the present resistance status of SXT^25,28^.

Further, our study revealed that the number of ICU patients with *S. maltophilia* infections increased during the COVID- 19 pandemic (2019, 2020, and 2021). Hence, our data contradict the conclusions drawn by the KFMC (2022) study and demonstrate a significant association between the occurrence of *S. maltophilia* isolates between patients in ICU and non-ICU settings before and during the COVID-19 pandemic. Globally, two studies conducted in China during the pandemic yielded data consistent with our own findings. These studies indicated that *S. maltophilia* was among the primary causes of coinfection among COVID-19 patients in the ICU. These findings suggest an increase in *S. maltophilia* infections during the pandemic period^29,30^. Once again, *S. maltophilia* has been demonstrated its role as an opportunistic pathogen in individuals with many chronic conditions and compromised immune systems.

Another key point, the Prophet package of R software was used to forecast the future number of isolates to be detected. Our findings showed a positive association between the number of *S. maltophilia* cases from 2004 to 2022 and the linear regression analysis. The coefficient of estimation (R^2^) was 0.65, suggesting a strong correlation. Additionally, the P value was < 0.001, further supporting the significance of the observed increase in the number of cases. According to this model, the rate of increase in *S. maltophilia* isolates is estimated to reach 15.08% [95% CI 12.58–17.59%] by 2030. As a result, it is imperative for health authorities worldwide, as well as in our region, to be vigilant and prepared to address this issue. The need for action is supported by several studies that have confirmed a concurrent increase in antibiotic resistance rates.

This study is valuable for two reasons. First, it represents pioneering research solely focused on *S. maltophilia* isolates and reaffirms prior observations regarding the escalating detection rate within 19 years, the concerning AMR rates, the effects of the COVID-19 pandemic, and the predicted future ascendancy of infection up to the year 2030. Second, when comparing the aforementioned rates with the global data, it becomes evident that the analysis of the most extensively reported number of *S. maltophilia* infections demonstrates significantly elevated levels of antibiotic resistance. However, this study has a few limitations that must be acknowledged. The data were collected over a long period (2004–2022); therefore, further analysis that involved classification by isolate type and patient subgroup was not possible. Additionally, the precise patterns of MDRs could not be monitored. The numbers of *S. maltophilia* isolate and AST findings may also have been affected by the various bacterial identification techniques employed from 2004 to the present day.

In conclusion, the antibiotic resistance rates of *S. maltophilia* are high and increasing. The alarmingly high rates of resistance to SXT (14.05%), LVX (56%), and ceftazidime (72.5%) are particularly concerning. According to the model used in this study, the prevalence of *S. maltophilia* detection is expected to increase continuously until 2030, which is supported by the present data. As a result, we encourage immediate measures to be taken on a global scale to address this growing issue with the assistance of healthcare authorities in establishing priorities and tracking the rise in infections and the scarcity of readily accessible treatments.

## Methods

### Study design

A 19-year retrospective investigation was conducted in King Faisal Specialist Hospital and Research Centre (KFSH&RC) in Riyadh, Saudi Arabia, from 2004 to 2022. The hospital has a bed capacity of 1549. KFSHRC collaborates with other 27 local hospitals for cooperative services. It also has active partnership agreements with Johns Hopkins Medicine International (JHMI) to improve the quality and safety of patient care. Hence, KFSHRC received samples from all over the country and most of the isolates represented various region.

### Data collection

A comprehensive collection of data from 4466 *S. maltophilia*-positive patients was obtained from diverse sources, encompassing blood samples, respiratory samples such as bronchoalveolar lavage and sputum, urine samples including indwelling catheters, as well as miscellaneous sources such as abscesses, wounds, tissues, and body fluids. All nonduplicated (separate) *S. maltophilia* isolates were included in this study. Therefore, each *S. maltophilia* isolation identified after 14 days was deemed to be a separate strain. Otherwise, all data of bacteria other than *S. maltophilia* were excluded, as well as any *S. maltophilia* strains that recovered from sources other than patients (e.g., hospital environment) for surveillance and/or infection control purposes.

### Identification and antimicrobial susceptibility testing of *S. maltophilia*

The identification and conducting susceptibility testing of *S. maltophilia* samples were carried out from 2004 to 2022. From 2004 to 2007, the identification of gram-negative bacilli (GNB) and non-glucose-fermenting gram-negative bacilli (NGNC) was performed using VITEK (bioMérieux, Marcy l’Etoile, France) ID-GNB and NGNC cards, as well as API 20 NE (bioMérieux, Marcy l’Etoile, France). In 2008, the identification methods used were VITEK2, Phoenix BD, and VITEK-MS™. Antimicrobial susceptibility testing (AST) was performed using automated systems to determine the MICs of various antibiotics. The MDR isolates were confirmed by E-test and broth microdilution techniques, in accordance with the recommended guidelines at that year. This testing was conducted exclusively on patient isolates that were conclusively confirmed to be *S. maltophilia*. The purpose was to evaluate the susceptibility of these isolates to ceftazidime (CAZ), trimethoprim-sulfamethoxazole (SXT), cefepime (FEP), levofloxacin (LVX), ciprofloxacin (CIP), gentamicin (GM), piperacillin-tazobactam (TZP), minocycline (MI), and ticarcillin-clavulanate (TIM). The findings were interpreted and documented in accordance with the Clinical and Laboratory Standards Institute (CLSI) rules for the assumed year and categorised as (susceptible [S], intermediate [I], or resistant [R]) and validated using an E-test.

### Statistical analysis

All collected data were stored and analysed using R version 4.1.2 (R Core Team, 2022) and GraphPad (version 9.0; PRISM). The study’s clinical data was summarized using descriptive statistics. Categorical variables were expressed as frequencies and percentages. Linear regression analysis was performed to understand the trends in *S. maltophilia* cases over time. We also used the R Prophet Package in R program to predict future cases up to 2030. This model forecasts time-series data based on an additive model, in where nonlinear trends fit the yearly patterns.

### Ethical considerations

The present study strictly adheres to the institutional guidelines that are mandatory for conducting research involving both humans and animals. Additionally, it fully complies with the relevant legislation and has obtained ethical approval. The Research Advisory Council (RAC) at King Faisal Specialist Hospital and Research Centre in Saudi Arabia has granted approval for this study (KFSH&RC: RAC #2230008). Furthermore, the Research Ethics Committee (REC) has waived the need for consent due to the retrospective nature of the study and the utilization of anonymized data. It is important to note that all aspects of the research were conducted in accordance with the principles outlined in the Declaration of Helsinki.

## Data Availability

The datasets produced and examined in the present investigation may be obtained from the corresponding author upon a reasonable request.

## References

[1] Adegoke AA, Stenström TA, Okoh AI (2017). *Stenotrophomonas maltophilia* as an emerging ubiquitous pathogen: Looking beyond contemporary antibiotic therapy. Front. Microbiol..

[2] Looney WJ, Narita M, Mühlemann K (2009). *Stenotrophomonas maltophilia*: An emerging opportunist human pathogen. Lancet Infect. Dis..

[3] Guerci P (2019). Outcomes of *Stenotrophomonas maltophilia* hospital-acquired pneumonia in intensive care unit: A nationwide retrospective study. Crit. Care..

[4] Brooke JS (2021). Advances in the microbiology of *Stenotrophomonas maltophilia*. Clin. Microbiol. Rev..

[5] Flores-Treviño S (2019). *Stenotrophomonas maltophilia* biofilm: Its role in infectious diseases. Expert Rev. Anti-infect. Ther..

[6] Majumdar R, Karthikeyan H, Senthilnathan V, Sugumar S (2022). Review on *Stenotrophomonas maltophilia*: An emerging multidrug-resistant opportunistic pathogen. Recent Patents Biotechnol..

[7] Brooke JS (2012). Stenotrophomonas maltophilia: An emerging global opportunistic pathogen. Clin. Microbiol. Rev..

[8] Çıkman A, Parlak M, Bayram Y, Güdücüoglu H, Berktas M (2016). Antibiotics resistance of *Stenotrophomonas maltophilia* strains isolated from various clinical specimens. Afr. Health. Sci..

[9] Zajac OM, Tyski S, Laudy AE (2022). Phenotypic and molecular characteristics of the mdr efflux pump gene-carrying *Stenotrophomonas maltophilia* strains isolated in warsaw, poland. Biology..

[10] Wu RX, Yu CM, Hsu ST, Wang CH (2022). Emergence of concurrent levofloxacin- and trimethoprim/sulfamethoxazole-resistant *Stenotrophomonas maltophilia*: Risk factors and antimicrobial sensitivity pattern analysis from a single medical center in taiwan. J. Microbiol. Immunol. Infect..

[11] Wang CH, Yu CM, Hsu ST, Wu RX (2020). Levofloxacin-resistant *Stenotrophomonas maltophilia*: risk factors and antibiotic susceptibility patterns in hospitalized patients. J. Hosp. Infect..

[12] Lai C-C, Chen S-Y, Ko W-C, Hsueh P-R (2021). Increased antimicrobial resistance during the covid-19 pandemic antimicrobial resistance antibiotic usage multidrug-resistant organism. Int. J. Antimicrob. Agents.

[13] Rehman, S. A parallel and silent emerging pandemic: Antimicrobial resistance (amr) amid covid-19 pandemic. 10.1016/j.jiph.2023.02.021 (2023). 10.1016/j.jiph.2023.02.021 PMC9942450 36857834

[14] CLSI. *Performance Standards for Antimicrobial Susceptibility Testing, M100*, 33 edn (Clinical and Laboratory Standards Institute, 2023). 10.1128/JCM.00213-21 PMC8601225 34550809

[15] Banar M (2023). Global prevalence and antibiotic resistance in clinical isolates of *Stenotrophomonas maltophilia*: A systematic review and meta-analysis. Front. Med..

[16] Naeem T, Absar M, Somily AM (2012). Antibiotic resistance among clinical isolates of *Stenotrophomonas maltophilia* at a teaching hospital in riyadh, saudi arabia. J. Ayub Med. Coll. Abbottabad JAMC.

[17] Hafiz TA (2022). *Stenotrophomonas maltophilia* epidemiology, resistance characteristics, and clinical outcomes: Under- standing of the recent three years’ trends. Microorganisms..

[18] Son HJ (2021). Risk factors for isolation of multi-drug resistant organisms in coronavirus disease 2019 pneumonia: A multicenter study. Am. J. Infect. Control..

[19] Raad M (2023). *Stenotrophomonas maltophilia* pneumonia in critical covid-19 patients. Sci. Rep..

[20] Alqahtani JM (2017). Emergence of *Stenotrophomonas maltophilia* nosocomial isolates in a saudi children’s hospital: Risk factors and clinical characteristics. Saudi Med. J..

[21] Dadashi M (2023). Global prevalence and distribution of antibiotic resistance among clinical isolates of *Stenotrophomonas maltophilia*: A systematic review and meta-analysis. J. Glob. Antimicrob. Resist..

[22] Dimopoulos G (2023). Upraising *Stenotrophomonas maltophilia* in critically ill patients: A new enemy?. Diagnostics..

[23] Wang N, Tang C, Wang L (2022). Risk factors for acquired Stenotrophomonas maltophilia pneumonia in intensive care unit: A systematic review and meta-analysis. Front. Med..

[24] Brooke JS (2014). New strategies against Stenotrophomonas maltophilia: A serious worldwide intrinsically drug-resistant opportunistic pathogen. Expert Rev. Anti-infect. Ther..

[25] Mojica MF (2022). Clinical challenges treating *Stenotrophomonas maltophilia* infections: An update. JAC-Antimicrob. Resist..

[26] Gil-Gil T, Martínez JL, Blanco P (2020). Mechanisms of antimicrobial resistance in Stenotrophomonas maltophilia: A review of current knowledge. Expert Rev. Anti-infect. Ther..

[27] Gibb J, Wong DW, Kritsotakis I, Karakonstantis S (2021). Antimicrobial treatment strategies for *Stenotrophomonas maltophilia*: A focus on novel therapies citation. Antibiotics.

[28] Khan A (2021). Evaluation of the performance of manual antimicrobial susceptibility testing methods and disk breakpoints for *Stenotrophomonas maltophilia*. Antimicrob. Agents Chemother..

[29] Sang L (2021). Secondary infection in severe and critical covid-19 patients in China: A multicenter retrospective study. Ann. Palliat. Med..

[30] Li J (2020). Etiology and antimicrobial resistance of secondary bacterial infections in patients hospitalized with covid-19 in Wuhan, China: A retrospective analysis. Antimicrob. Resist. Infect. Control..

